# Chinese patients’ clinical and psychosocial outcomes in the 6 months following percutaneous coronary intervention

**DOI:** 10.1186/s12872-021-01954-2

**Published:** 2021-03-23

**Authors:** Xia Liu, Adeleke Fowokan, Sherry L. Grace, Biao Ding, Shu Meng, Xiu Chen, Yinghua Xia, Yaqing Zhang

**Affiliations:** 1 Shanghai Jiao Tong University School of Nursing, Shanghai, China; 2 KITE-Toronto Rehabilitation Institute and Peter Munk Cardiac Centre, University Health Network, University of Toronto, Toronto, ON Canada; 3 Faculty of Health, York University, Bethune 368, 4700 Keele Street, Toronto, ON M3J 1P3 Canada; 4 Shanghai Sixth People’s Hospital, Shanghai, China; 5 Xinhua Hospital Affiliated to Shanghai Jiaotong University School of Medicine, Shanghai, China

**Keywords:** Angina, Anxiety disorders, Cardiovascular diseases, Cardiovascular risk factors, China, Depression, Percutaneous coronary intervention, Quality of life, Signs and symptoms, Sleep

## Abstract

**Background:**

In China, there has been a precipitous increase in the number of percutaneous coronary interventions (PCI) conducted. We sought to characterize the clinical and psychosocial trajectory of PCI patients from the time of procedure through 6 months post, and correlates of adverse cardiovascular events (ACEs).

**Methods:**

In this prospective, observational study, patients from 2 hospitals in Shanghai, China were assessed. At follow-up visits at 1, 3 and 6 months post-PCI, clinical indicators were again extracted from patients’ clinical records, including ACEs, and they completed validated surveys assessing self-management, as well as psychosocial indicators (Hospital Anxiety and Depression Scale; Pittsburgh Sleep Quality Index; quality of life [QoL]: SF-12, Seattle Angina Questionnaire [SAQ]). Repeated measures analysis of variance, adjusted for Barthel index and PCI indication, was used to assess change over time in risk factors and psychosocial indicators. Logistic regression was used to explore correlates of ACEs.

**Results:**

610 participants (mean age = 63.3; n = 150, 18.2% female) were recruited, of which 491 (80.5%) were retained at 6 months. 82 (16.7%) had an ACE at any time point, including most commonly angina and stroke (only 1 death). Clinical indicators such as blood pressure (p < 0.031 for both), symptom burden (p < .01 on all subscales) and QoL (p < 0.001 for both, but started quite low) improved over 6 months. Anxiety and depressive symptoms were above threshold, and the latter worsened over time (p < 0.001). With adjustment for age and indication, patients with any ACEs had higher sleep latency (odds ratio [OR] = 1.48; 95% confidence interval [CI] = 1.03–2.10]), and depressive symptoms (OR = 1.20; 95% CI = 1.02–1.41), but lower anxiety (OR = 0.79; 95% CI = 0.67–0.93) compared to those without.

**Conclusion:**

Centers may wish to re-visit patient selection criteria and processes for PCI, as well as implement mental health screening and treatment protocols, as can be achieved through cardiac rehabilitation, given how hazardous psychosocial distress is in this population.

## Introduction

Cardiovascular diseases (CVD) are among the leading causes of morbidity and mortality globally [1], with a particularly high burden in lower-resource countries such as China [2]. Indeed, between 1990 and 2016, the prevalence of CVDs rose from approximately 40.6 million to 93.8 million there [2]. 

One of the most common acute CVD treatment modalities is percutaneous coronary intervention (PCI). Indeed, an estimated 400,000 PCI procedures are now conducted every year in China, despite their lower resources [3] (versus ~ 600,000 in the United States for example) [4]. The high rate [3] of PCIs performed has led to concerns about the appropriateness, risks and benefits of these procedures in patients who undergo them [5]. Indeed, in one study which tracked 1600 Chinese patients who underwent PCI for stable coronary artery disease, they found that 26% of the patients had no pre-procedural angina symptoms [6].

Following PCI, patients need to continue to reduce their risk through secondary prevention strategies [7]; it is known that few patients reach the recommended targets for the multiple risk factors (e.g., blood pressure, heart-health behaviors) [8], and most do not access cardiac rehabilitation (CR) to support them in doing so [9].

However, there have not been many studies, including in lower-resource settings, where cardiac and/or PCI outpatients are followed to determine their rate of adverse cardiovascular events (ACE), risk factor control and quality of life (QoL), including angina symptoms [5, 6, 10]. We know that risk of recurrent events is even higher in those with CVD who also suffer from psychosocial distress such as depression and anxiety as well as sleep issues [11, 12], and this type of distress can be higher in China [13].

Therefore, the objectives of this study were to characterize the clinical and psychosocial trajectory of PCI patients in China through 6 months post-procedure. The primary outcome was ACEs (mortality, morbidity), while secondary outcomes included CVD risk factor management (e.g., hypertension, tobacco use, and physical inactivity), and psychosocial well-being (QoL including angina symptoms, anxiety, depressive symptoms and sleep quality). Second, clinical and psychosocial correlates of ACEs were explored.

## Methods

### Design, setting and procedure

This was a longitudinal, observational study. The study was approved by the Xinhua Hospital Research Ethics Board. PCI patients at the Xinhua Hospital affiliated with Shanghai Jiaotong University School of Medicine and Shanghai Sixth People’s Hospital were recruited between December, 2016 and November 2018. Neither center has a CR program.

The research nurse at each hospital approached patients during hospitalization to invite them to participate (informed consent). Data were collected at 4 points: (1) during hospitalization (after PCI but before discharge), (2) 1 month (± 3 days) post-discharge, (3) 3 months (± 1 week) post-discharge, and (4) 6 months (± 1 week) post-discharge. Data were collected from the electronic medical records and through on-site interviews in Mandarin, including during clinical follow-up visits post-discharge. Specifically, after discharge, patients were seen regularly at a clinic for follow-up by the physician who did the PCI procedure. When the patients were coming for the appointment closest to the time of their next scheduled research follow-up, the research nurse would call the patients to invite them to come earlier to allow for data collection. Patients were then provided questionnaires to assess outcomes of interest, including psychosocial well-being and ACEs. For patients who missed post-discharge clinic appointments, telephone interviews were used to obtain follow-up data.

### Participants

Inclusion criteria were: age 18–75 years, hospitalized for PCI, planning to receive follow-up care from same institution, and sufficient ability to understand the assessment questions and communicate responses. Exclusion criteria were: patient history of serious mental illness (self-reported to resident), severe arrhythmia, heart failure, cardiogenic shock or other life-threatening conditions, and failed PCI.

### Measures

At recruitment, clinical characteristics of patients were extracted from electronic medical records including cardiac history, cardiac risk factors, disease severity indicators, characteristics of the PCI (including length of stay), cardiac medications and blood work results. The Barthel index, [19] which characterizes activities of daily living, was extracted from the electronic patient record.

ACEs included the following: death (any cause), re-hospitalization (cardiac-related), myocardial infarction (non-fatal), stroke, heart failure (HF) development or exacerbation, PCI-related complications (in-stent restenosis, stent thrombosis or major bleeding event), peripheral vascular embolism, recurrent angina, among others (i.e., arrhythmias, coronary revascularization and transient ischemic attacks). ACEs were ascertained through the self-report questionnaire (for events occurring outside the centre) and from electronic patient medical records at each assessment point.

**Fig. 1:**
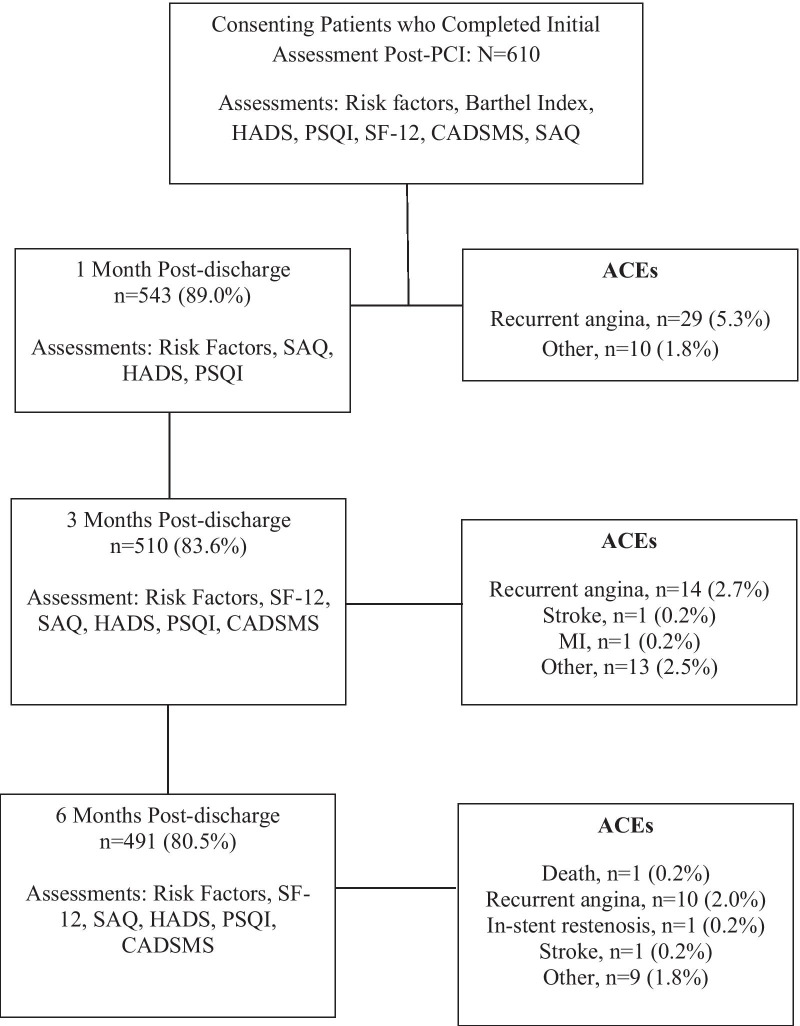
Participant flow diagram, with ACEs. BP = Blood Pressure, SAQ = Seattle Angina Questionnaire, HADS = Hospital Anxiety and Depression Scale, PSQI = Pittsburgh Sleep Quality Index, CADSMS = Coronary Artery Disease Self-Management Scale, PCI = Percutaneous Coronary Intervention, ACE = Adverse Cardiovascular Events, SF-12: Short-Form quality of life survey (Medical Outcomes Study), MI = Myocardial Infarction

Risk factors assessed at each time point included blood pressure, which was assessed by a trained nurse using a sphygmomanometer. Lipids (total cholesterol [TC], triglycerides [TG], low-density lipoprotein [LDL] and high-density lipoprotein [HDL] were assessed at the point of admission into hospital and after 6 months using a blood test. Additionally, patient self-reported tobacco use, alcohol use, physical activity (duration, intensity, and type) and medication adherence (patients were asked if they take their medication regularly or not; measured on 5-pt Likert-type scale) were assessed at most assessment points. The psychometrically-validated Coronary Artery Disease Self-Management scale (CADSMS) [14] was also administered at all but the 2^nd^ assessment (Fig. 1). It consists of 27 items in 7 areas: leisure management, symptom management, emotional/ cognitive management, first aid education, disease knowledge, daily life management, and treatment compliance. Response options are on a 5-point Likert scale; The item scores for each subscale are summed. A total score is also computed, ranging from 27 to 135 points, with higher scores indicating better individual self-management behavior. The CADSMS scale has good retest reliability (intra-class correlation = 0.910), internal consistency (Cronbach’s α = 0.913), and validity [15].

Psychometrically-validated, translated scales were also administered at each assessment point to assess psychosocial well-being (Fig. 1). The Seattle Angina Questionnaire [16] (SAQ; physical limitation, angina stability, angina frequency, treatment satisfaction, disease perception subscales), Hospital Anxiety and Depression Scale [17] (HADS), and the Pittsburgh Sleep Quality Index [18] (PSQI; 7 subscales) were assessed at each point. The SF-12 (QoL) [19] was administered at 3 of the 4 assessment points only: in-hospital as well as at 3 and 6 months post-discharge. All 4 scales are shown to be reliable and valid [16–19].

### Statistical analyses

Statistical Package for Social Sciences (SPSS v.26) was used for analyses. A p-value < 0.05 was used to denote statistical significance. First, differences in the sociodemographic and clinical characteristics of patients retained at 6 months post-discharge were compared to those lost to follow-up using t-tests or chi-square (Fisher’s exact test where cell sizes small) as applicable.

ACEs, risk factors and psychosocial indicators were described at each assessment point, and change over time in the latter 2 assessed using repeated measures analysis of variance (ANOVA), with adjustment for PCI indication and Barthel index. Finally, clinical and psychosocial correlates of ACEs were investigated. First, differences in the sociodemographic, clinical and psychosocial characteristics at the initial assessment point of patients with and without ACEs were compared using independent samples t-tests and chi-squared analysis, as applicable. Variables that were found to be significantly associated with ACEs in these bivariate analyses were then included in a multivariate logistic regression, to test their association with ACEs, excluding those with indication of multicollinearity.

## Results

### Respondent characteristics

As shown in Fig. 1, there were 610 consenting patients; their characteristics are shown in Table 1. Two hundred and sixty-eight patients (43.9%) had a body mass index > 25 at the time of the PCI. Barthel index scores indicated that study participants were able to complete daily activities independently, exhibiting only minimal dysfunction. Almost half the sample received PCI without confirmed acute coronary syndrome (ACS).

**Table 1:** Sociodemographic and clinical characteristics of participants by retention status

Characteristics	Total (N = 610)	Retained at 6 months (n = 491)	Lost to follow-up (n = 119)	p*	p^†^
**Sociodemographic**					
Age (years)	62.3 ± 11.1	62.4 ± 11.1	62.0 ± 10.8	0.729	0.047
Sex (% Male)	460 (75.4%)	365 (74.3%)	95 (79.7%)	0.212	0.332
Nationality (% Han)	605 (99.3%)	487 (99.2%)	118 (99.2%)	0.325	0.658
*Insurance*				0.006	0.137
Employee medical insurance	251 (41.2%)	215 (43.8%)	36 (30.5%)		
Urban residents’ medical insurance	296 (48.6%)	223 (45.4%)	73 (61.9%)		
Public or rural cooperative medical care	12 (1.9%)	8 (1.6%)	4 (3.4%)		
Self-pay	50 (8.2%)	45 (9.2%)	5 (4.2%)		
**Clinical**					
*Cardiac history*					
Previous MI	44 (7.2%)	28 (5.7%)	16 (13.4%)	0.001	0.708
Previous HF	6 (1.0%)	2 (0.4%)	4 (3.4%)	0.004	0.555
Previous valve procedure	3 (0.5%)	2 (0.4%)	1 (0.8%)	0.053	0.840
Previous PCI	82 (13.4%)	55 (11.2%)	27 (22.7%)	< 0.001	0.602
Previous CABG	2 (0.3%)	2 (0.4%)	0	0.074	0.880
*Comorbidities*					
Cerebrovascular Disease	50 (8.6%)	40 (8.1%)	10 (8.4%)	0.126	0.199
Kidney disease requiring dialysis	1 (0.2%)	0	1 (0.8%)	0.038	0.846
Peripheral arterial disease	0	0	0	–	0.683
Lung disease	18 (3.0%)	11 (2.2%)	7 (5.9%)	0.013	0.196
***PCI information***					
*Indication for PCI*				< 0.001	< 0.001
Acute coronary syndrome	318 (52.1%)	287 (58.9%)	31 (26.5%)		
Atherosclerotic lesions in coronary arteries without ACS	284 (46.6%)	198 (40.7%)	86 (73.5%)		
*Culprit vessel*				< 0.001	0.001
Left main	5 (0.8%)	3 (0.6%)	2 (1.7%)		
Right coronary artery	105 (17.2%)	76 (15.5%)	29 (24.4%)		
Left circumflex	91 (14.9%)	63 (12.8%)	28 (23.5%)		
Left anterior descending	208 (34.1%)	163 (33.2%)	45 (37.8%)		
More than 2 vascular lesions	201 (33.0%)	186 (37.9%)	15 (12.6%)		
*Other*					
Degree of stenosis	91.6 ± 12.8	92.2 ± 12.6	89.5 ± 13.7	0.060	< 0.001
Troponin	2.0 ± 7.6	2.0 ± 8.2	1.7 ± 4.2	0.656	0.202
D-dimer	2.7 ± 4.3	4.5 ± 0.2	0.6 ± 0.1	< 0.001	0.001
***Risk factors***					
Hypertension	396 (64.9%)	315 (64.2%)	81 (68.1%)	0.655	0.915
Dyslipidemia	224 (36.7%)	172 (35.0%)	52 (43.7%)	0.078	0.146
Body mass index	24.8 ± 3.4	24.9 ± 3.4	24.8 ± 3.3	0.729	0.297
Smoking in past year	230 (37.7%)	185 (37.7%)	45 (37.8%)	0.978	0.445
Family history of premature onset CVD	174 (28.5%)	142 (28.9%)	32 (26.9%)	0.117	0.821
*Diabetes*	195 (32.0%)	156 (31.8%)	39 (32.8%)	0.834	0.344
Fasting blood glucose	7.1 ± 3.5	7.1 ± 3.7	6.7 ± 2.4	0.332	0.978
HbA1_c_	6.5 ± 3.1	6.4 ± 3.4	6.6 ± 1.5	0.626	0.166
C-reactive protein	10.7 ± 66.8	12.1 ± 74.6	5.5 ± 15.1	0.410	0.514
***Disease severity indicators/other clinical factors***					
Heart rate (bpm)	74.5 ± 11.2	74.5 ± 11.5	74.5 ± 10.2	0.969	0.090
*Killip class*				< 0.001	0.840
Class 1	368 (60.3%)	327 (90.3%)	41 (50.6%)		
Class II	61 (10.0%)	26 (7.2%)	35 (43.2%)		
Class III	2 (0.3%)	2 (0.5%)	0		
Barthel index^a^	81.0 ± 23.7	78.1 ± 24.7	92.3 ± 13.6	0.007	< 0.001
Ejection fraction	31.4 ± 71.6	29.3 ± 74.2	55.8 ± 18.8	< 0.001	< 0.001
BNP	291.0 ± 906.7	220.8 ± 579.6	622.1 ± 1734.3	< 0.001	0.013
Length of stay (days)	6.6 ± 3.3	6.5 ± 3.1	6.9 ± 3.9	0.262	0.066
*Medications*					
Anti-platelets	605 (99.2%)	487 (99.2%)	118 (99.2%)	0.545	0.278
Lipid-lowering	580 (95.1%)	477 (97.2%)	103 (86.6%)	< 0.001	0.295
Anti-hypertensives	505 (82.9%)	414 (84.3%)	91 (77.1%)	< 0.001	0.002
ACE inhibitors	278 (45.6%)	226 (46.0%)	52 (43.7%)	0.214	< 0.001
Anti-arrhythmics	31 (5.1%)	23 (4.7%)	8 (6.7%)	0.251	0.154
Proton pump inhibitors	143 (23.4%)	107 (21.8%)	36 (30.3%)	< 0.001	< 0.001

n and % (based on available data; some was missing) or mean ± standard deviation shown

BMI = body mass index, BP = blood pressure, HADS = Hospital Anxiety and Depression scale, TG = triglycerides, TC = total cholesterol, LDL = low-density lipoprotein, HDL = high-density lipoprotein, FBG = fasting blood glucose, CRP = C-reactive protein, A1c = glycated hemoglobin, EF = ejection fraction, PCI = percutaneous coronary intervention, CABG = coronary artery bypass graft, CAD = coronary artery disease, BNP = B-type natriuretic peptide, MI = myocardial Infarction, HF = heart failure, BPM = beats per minute, CKMB = creatine kinase-MB, CVD = cardiovascular disease, ACS = acute coronary syndrome

*p for comparison of variable by 6-month follow-up retention (yes/no), using chi-square (fisher’s exact with small cell sizes) or t-test, as appropriate

^†^p for association with adverse cardiovascular events at 6 months (any; yes vs no), tested using independent samples t-test or chi-square analysis, as applicable

^a^Scores range from 0 to 100, with higher scores indicating better ability to engage in activities of daily living

Of these, 491 (80.5%) patients were retained at the final 6-month assessment (Fig. 1). Differences in patients’ characteristics at the time of PCI by follow-up status are also presented in Table 1. Patients who completed follow-up had significantly different medical insurance sources, were less likely to have had previous cardiac events and PCI, less likely to have comorbid kidney and lung disease, were more likely to have had confirmed ACS as their indication for the PCI, had different culprit vessels, higher D-dimer, had lower Killip classification, ejection fraction and brain natriuretic peptide, were less able to engage in activities of daily living (Barthel Index), as well as used more lipid-lowering and anti-hypertensive medications (but less proton pump inhibitors) than those lost to follow-up. No other differences were observed.

### Outcomes over time

ACEs are shown in Fig. 1; There was only 1 death. Among those retained at 6 months, over the 6 months, 82 (16.7%) participants had any ACE at any time point, which was most commonly recurrent angina.

Risk factors and their management are shown in Table 2. Systolic BP went down significantly over time. As shown, at 6 months post-PCI, most patients were at guideline target. Patients’ salt intake, and fried food consumption decreased significantly over the 6-month period. Additionally, physical activity and medication adherence increased significantly. Lastly, there were significant increases across all subscales of the CADSMS except emotional/cognitive management, indicating improved risk factor self-management.

**Table 2:** CVD risk management over 6 months from the percutaneous coronary intervention, n = 491

Measure	Post-PCI	1st month	3rd month	6th month	p^ǁ^
*Blood pressure (mmHg)*					
Systolic	128.9 ± 19.1	123.8 ± 14.6	124.2 ± 13.1	124.7 ± 14.9	0.043
% over 140 systolic	70 (14.3%)	34 (6.9%)	24 (4.9%)	31 (6.3%)	
Diastolic	75.3 ± 10.8	73.6 ± 9.1	73.6 ± 8.2	74.3 ± 7.8	0.125
*Tobacco use* (% current)	202 (41.2%)	–	134 (27.3%)	129 (26.3%)	0.588^§^
*Harmful use of alcohol*^a^					0.157
Each time > 60 ml pure alcohol (beer > 520 ml, wine > 180 ml, Chinese spirits > 45 ml)	33 (6.7%)	–	8 (1.6%)	12 (2.4%)	
Each time 40 ~ 60 ml pure alcohol (350–520 ml beer, 120–180 ml wine, Chinese spirits 30–45 ml)	11 (2.2%)	–	4 (0.8%)	1 (0.2%)	
Each time < 40 ml pure alcohol (350 ml beer, 120 ml wine, 30 ml Chinese spirits) and often drinking	18 (3.7%)	–	8 (1.6%)	11 (2.2%)	
< 40 ml pure alcohol and occasionally drinking	117 (23.8%)	–	99 (20.2%)	90 (18.3%)	
Never drink alcohol	312 (63.5%)	–	372 (75.8%)	377 (76.8%)	
*Monthly salt consumption*^a,b^					< 0.001
< 120 g	78 (15.9%)	–	89 (18.1%)	98 (20.0%)	
120–149 g	135 (27.5%)	–	214 (43.6%)	256 (52.1%)	
150–179 g	127 (25.9%)	–	143 (29.1%)	94 (19.1%)	
180–210 g	101 (20.6%)	–	37 (7.5%)	32 (6.5%)	
> 210 g	50 (10.2%)	–	8 (1.6%)	10 (2%)	
*Fried food intake (weekly)*^a^					< 0.001
Not consumed	316 (64.4%)	–	382 (77.8%)	389 (79.2%)	
1–4 servings	163 (33.2%)	–	106 (21.6%)	97 (19.8%)	
5–7 servings	10 (2.0%)	–	3 (0.6%)	4 (0.8%)	
> 7 servings	2 (0.4%)	–	0	1 (0.2%)	
*Physical activity (minutes moderate to vigorous intensity/week)*^a^					0.040
None	84 (17.1%)	–	68 (13.8%)	87 (17.7%)	
< 30	99 (20.2%)	–	89 (18.1%)	70 (14.3%)	
30–60	85 (17.3%)	–	74 (15.1%)	57 (11.6%)	
60–180^c^	67 (13.6%)	–	108 (22.0%)	93 (18.9%)	
> 180	156 (34.2%)	–	152 (31.0%)	184 (37.5%)	
*Exercise type*					
None	–	286 (58.2%)	60 (12.2%)	55 (11.2%)	0.018^§^
Walk	–	157 (32.0%)	194 (39.5%)	190 (38.7%)	
Jog	–	3 (0.6%)	7 (1.4%)	10 (2.0%)	
Tai Chi	–	1 (0.2%)	4 (0.8%)	4 (0.8%)	
*Medication adherence*					< 0.001
Never	51 (10.4%)	–	6 (1.2%)	7 (1.4%)	
Hardly	26 (5.3%)	–	8 (1.6%)	15 (3.1%)	
Sometimes	33 (6.7%)	–	25 (5.1%)	30 (6.1%)	
Often	80 (16.3%)	–	96 (19.6%)	106 (21.6%)	
Always	301 (61.3%)	–	354 (72.1%)	330 (67.2%)	
*CAD self-management*^a^					
Leisure activity management	13.6 ± 3.2	–	15.5 ± 2.4	15.7 ± 2.4	< 0.001
Symptom management	9.1 ± 3.5	–	10.6 ± 3.3	11.0 ± 3.5	< 0.001
Emotional/cognitive management	13.7 ± 2.8	–	14.1 ± 3.0	14.4 ± 3.0	0.055
First aid	7.5 ± 3.3	–	9.0 ± 3.1	9.4 ± 3.2	< 0.001
Disease knowledge	13.0 ± 4.2	–	14.7 ± 3.6	14.7 ± 3.6	< 0.001
Daily life management	14.6 ± 3.0	–	15.4 ± 2.7	15.5 ± 3.0	0.037
Treatment adherence	6.1 ± 2.1	–	7.5 ± 1.6	7.3 ± 1.7	< 0.001
CADSMS total	77.5 ± 13.7	–	86.8 ± 11.5	87.9 ± 11.3	< 0.001

Mean ± standard deviation or n (%) shown

BP = blood pressure, CAD = coronary artery disease

^a^From CADSMS (Coronary Artery Disease Self-Management Scale [14])

^b^Recommended daily intake 2.0grams, so monthly would be ~ 60 [33]

^c^Recommended weekly amount = 150 min moderate to vigorous intensity [34]

–Not assessed at this time point

^§^Chi-square test comparing first to last assessment for categorical variables

^ǁ^Adjusted for percutaneous coronary intervention indication and Barthel index

**Fig. 2:**
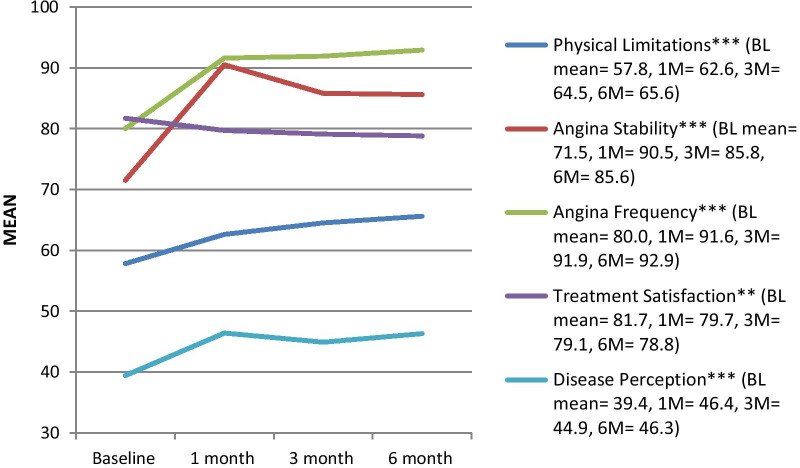
SAQ subscale scores over the 6 months from the percutaneous coronary intervention, n = 491. SAQ = Seattle Angina Questionnaire [16], BL = Baseline (post-PCI), M = month. *Note*: The SAQ has five subscales, with higher scores being more positive. Subscale scores range from 0 to 100. No overall scale score is generated. To facilitate clinical interpretability, ranges of SAQ Angina Frequency subscale scores can be translated qualitatively into daily (0–30), weekly (31–60), monthly (61–99), and no (100) angina. The SAQ QoL subscale scores can be translated into very poor to poor (0–24), fair (25–49), good (50–74), and excellent (75–100) quality of life. As per previous work, we considered an increase in the SAQ subscale scores of 10 or more points as clinically significant improvement [6]. *p < 0.05, **p < .01, ***p < 0.001

QoL indicators are shown in Table 3 and Fig. 2. The physical composite health score of the SF-12 was low at time of PCI, and increased significantly over time. Similarly, significant differences were observed with regard to symptoms over the 6 months, with all SAQ subscales increasing from the time of the PCI (it appeared the greatest increases occurred in the first month) but the treatment satisfaction subscale. Increases on the anginal stability and frequency subscales could be considered clinically meaningful (Fig. 2). Angina symptoms were good to excellent throughout the 6-month trajectory.

**Table 3:** Psychosocial indicators over the 6 months from the percutaneous coronary intervention, n = 491

Measures	Baseline	1st month	3rd month	6th month	p^ǁ^
**SF-12**^a^					
Physical health	30.6 ± 9.7	–	32.8 ± 9.2	33.7 ± 9.8	0.004
Mental health	36.4 ± 8.1	–	37.7 ± 7.3	38.7 ± 7.8	0.566
Overall	67.0 ± 16.3	–	70.5 ± 15.3	72.4 ± 16.6	0.216
HADS*					
Elevated depressive symptoms	349 (71.1%)	378 (76.9%)	392 (79.9%)	404 (82.3%)	
Elevated anxiety symptoms	483 (98.4%)	486 (99.0%)	480 (97.8%)	481 (98.0%)	
**PSQI total**	6.2 ± 3.4	6.0 ± 3.3	5.6 ± 3.0	5.5 ± 2.9	0.432

–Not assessed at this point

* > 7

^a^Scores range from 0 to 100, with 0 representing worse health and 100 best health. Normative scores from the US are 50 ± 10 [35]

^ǁ^Adjusted for percutaneous coronary intervention indication and Barthel index

PSQI = Pittsburgh Sleep Quality Index; HADS = Hospital Anxiety and Depression Scale; SF-12 = short-form (quality of life)

**Fig. 3:**
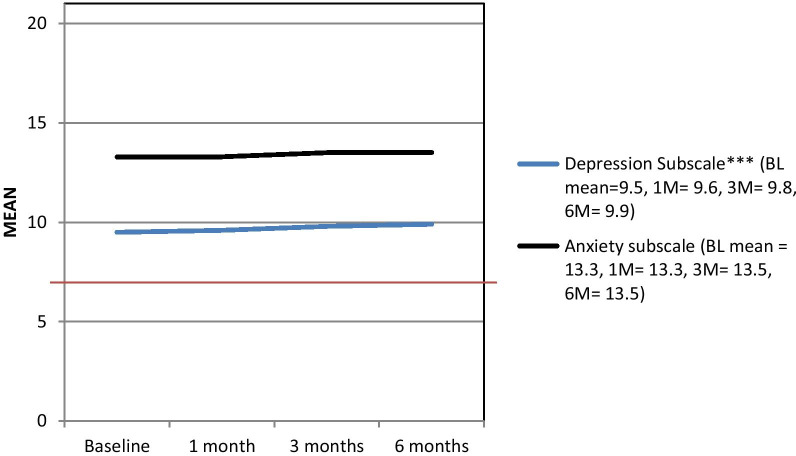
HADS subscale scores over the 6-month follow-up from the percutaneous coronary intervention, n = 491. HADS = Hospital Anxiety and Depression Scale [17], BL = Baseline, M = month. *Note*: The HADS has two subscales: Anxiety and Depressive symptoms. Subscale scores range from 0–21; Higher scores indicate higher anxiety or depressive symptoms. Scores above 7 indicate “elevated” symptoms. *p < .05, **p < 0.01, ***p < 0.001

**Fig. 4:**
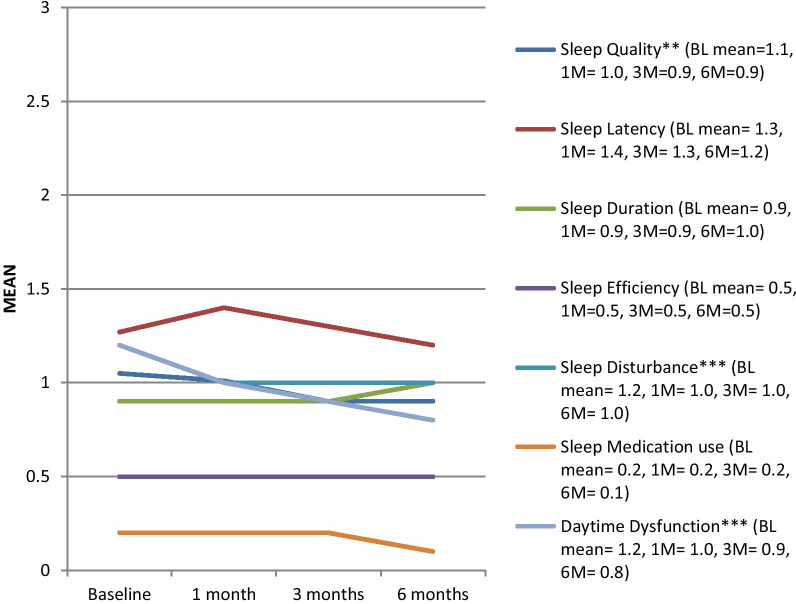
PSQI subscale scores over the 6-month follow-up from the percutaneous coronary intervention, n = 491. PSQI = Pittsburgh Sleep Quality Index [18], BL = Baseline, M = month. *Note*: The PSQI has 7 subscales. Subscale scores range from 0 (no difficulty) to 3 (severe difficulty); Higher scores indicate worse sleep quality. *p < .05, **p < 0.01, ***p < 0.001

Other psychosocial well-being indicators are shown in Figs. 3 and 4, as well as Table 3. Distress was high (particularly anxiety), and still depressive symptoms were found to significantly increase from the time of PCI (anxiety remained consistently high). On the contrary, 4 of the PSQI subscales – sleep quality (p = 0.003), sleep latency (p = 0.041), sleep disturbance (p < 0.001), and daytime dysfunction (p < 0.001) were found to decrease significantly over 6 months, indicating improved sleep quality (change in total score did not sustain adjustment; Table 3).

### Correlates of ACEs

Patients who had any ACE over the 6 months were significantly more likely to be older, have had a confirmed ACS (and accordingly had a higher degree of stenosis, particularly in the left main artery), higher d-dimer, and to be on ACE-inhibitor and anti-hypertensive medications than those who did not at time of PCI. They also had lower Barthel index scores, ejection fraction, b-type natriuretic peptide levels, and were less likely to be on proton pump treatment than those who did not (Table 1).

With regard to associations with psychosocial indicators at 6 months, those with an ACE at any point had significantly greater depressive symptoms (p < 0.001), and significantly less anxiety (p < 0.001). Those who had any ACE at any time point also had poorer physical (p < 0.001) and mental (p < 0.001) QoL. With regard to the SAQ, those who had an ACE also had significantly greater physical limitations (p = 0.010), angina frequency (p = 0.007), and poorer disease perceptions (p < 0.001). They had poorer sleep quality (daytime dysfunction [p = 0.020], sleep disturbance [p < 0.001] and sleep latency [p < 0.001]). Finally, those with an ACE had poorer emotional/cognitive self-management of their CAD (p < 0.001) and first aid knowledge (p = 0.008), but better symptom management (p < 0.001) than those who had no ACE at any point. No other differences were observed.

In logistic regression analyses assessing correlates of ACEs with ten significant independent variables from the above bivariate analyses (i.e., age, PCI indication, physical QoL, anxiety symptoms, depression symptoms, sleep disturbance, sleep latency, emotional/cognitive management, disease perception and anginal frequency), higher age (odds ratio [OR] = 1.029 [95% confidence interval [CI]: 1.00 – 1.06], p = 0.045), higher sleep latency (OR = 1.48 [95% CI: 1.03 – 2.10], p = 0.032), and depressive symptoms (OR = 1.20 [95% CI: 1.02 – 1.41], p = 0.024), as well as lower anxiety (OR = 0.79 [95% CI: 0.67 – 0.93], p = 0.005) survived this adjustment.

## Discussion

This study provides insight into the nature of, as well as clinical and psychosocial trajectory of Chinese patients over 6 months post-PCI. Half of the cohort did not have an ACS prior to PCI, and the burden of angina symptoms was not high. For over 10% of the cohort, this was not the first PCI. Many patients did not have a history of heart disease. As is established, cerebrovascular disease was quite common. Rates of hypertension were high, but blood pressure was well-controlled. Rates of smoking and diabetes were quite high, and harmful use of alcohol low. Length of stay was ~ 6.5 days. Use of anti-platelets and lipid-lowering medication was high, and patients were highly adherent.

Major ACEs were not common; the major patient issue was recurrent angina, but SAQ scores reveal the overall burden of angina in most patients in the cohort was not high. This is likely a reflection of the cohort as outlined above, and also due to the improvements patients made in their heart-health behaviour post-PCI. It is unfortunate that rates of PCI are higher than use of CR, which is much cheaper and often equally effective [20].

Worrisome was the extraordinarily high burden of depressive and anxiety symptoms, and QoL was quite low. Indeed, depressive symptoms and anxiety started above the threshold that may be indicative of a severity warranting diagnosis, and depressive symptoms significantly worsened with time; QoL did improve somewhat, certainly not to a clinically meaningful degree, but remained quite a bit lower than what we see in Western cohorts [21]. Given these patients did appear to have frequent follow-up visits, while it appears risk factors and clinical issues are well-addressed, capacity for mental health screening and care should be increased in the cardiac setting in China. Again, this can often be addressed in CR, as long as programs have sufficient resources to hire a mental healthcare provider as part of the team [22]. Patients should be screened, and provided evidence-based treatment options including pharmacotherapy and psychotherapy, which should be stepped, and with good follow-up care, where warranted [23]. These psychosocial issues will in time likely impact patient’s clinical health status and longevity if left untreated.

Findings from this study appear consistent with results from the few studies conducted in China and other countries [24]. Schmidt et al. found high prevalence of anxiety, stress and depression in Brazilian patients who recently underwent PCI [25]. Similarly, in one longitudinal study from China, high rates of post-PCI anxiety and depression were observed; additionally, patients who had depression had higher rates of ACE, including cardiac-related mortality and revascularization during the 3-year follow-up period [9]. In one systematic review, improvements in all subscales of the SF-12 and SAQ were observed following PCI [26]. These findings are consistent with our findings, with the exception of treatment satisfaction which was lower with outpatient than inpatient care.

ACEs were associated with greater depressive symptoms as expected [11], but as has been found in other studies [27], anxiety likely incited better self-management as it was associated with less ACEs. The impact of anxiety severity on outcomes warrants further study. Poor sleep was hazardous, consistent with other studies [28]. Angina symptoms were clearly indicative of poor coronary circulation, and ability to control those symptoms protective. Better QoL was protective (likely reverse causality), so the fact that it was quite low in this cohort is worrisome.

There are several mechanisms which can explain the association between ACEs and psychosocial distress [29–31]. Increased levels of psychosocial distress might alter the neuro-endocrine system via the activation of the sympathetic nervous system [32]. This in-turn might result in a cascade of events, including endothelial dysfunction, production of pro-inflammatory cytokines etc. that could affect the cardiovascular system [32].

The high rate of anxiety, and it’s association with less ACEs in this cohort suggesting they are engaging in health-protective behaviours, raises the question of whether perhaps these anxious patients are seeking healthcare to a greater degree than the average patient (i.e., the so-called “worried well”). Indeed, many of the patients had a PCI without an ACS (appropriateness cannot be determined from the data available). This would require further investigation, given anxiety rates in non-participants are unknown. But this does raise questions about PCI selection processes, and again potentially the need for more mental health screening.

### Study limitations

Caution is warranted in interpreting these findings. First, this was a convenience sample; as such, it is unknown whether there could be selection bias in the sample, particularly considering response rate was not tracked. For information, Xinhua Hospital performs approximately 1500 PCIs per year. Indeed, second, generalizability is also limited for a few reasons. They are limited to patients in Shanghai; whether findings are applicable to other cities or regions of China is unknown. Moreover, participants were primarily male. Third, there was some retention bias in the sample, further limiting the generalizability of findings from the latter assessments. Fourth, sample size was not calculated a priori as we sought to observe clinical and psychosocial trajectory in the cohort; however considering the rule of thumb of 10 participants per variable, sample size should be considered sufficient for the second objective (i.e., we did not test 50 ACE correlates). Fifth, only 1 ACE was recorded at each time point, and was missing for some participants. Sixth, multiple comparisons were undertaken, inflating the possibility of error. Last, due to the nature of the study design, causal conclusions cannot be drawn.

In conclusion, through this study the nature and trajectory of PCI patients in a major Chinese city, where rates of these procedures are exploding, was explored. Just over 15% of patients had any ACE, and this was most often angina; However, overall anginal burden was not high, and improved meaningfully over time. Patient self-management and risk factor control were good, although tobacco use, salt and physical activity require more attention. Patients were highly anxious and depressed, and this did not improve with time. QoL was quite low. Results suggest centers may wish to re-visit patient selection criteria and processes for PCI, as well as implement mental health screening and treatment protocols, as can be achieved through CR.

## Data Availability

The dataset used for the current study is available from the corresponding author on reasonable request.

## Abbreviations

ACE: Adverse cardiovascular events
ACS: Acute coronary syndrome
BNP: B-type natriuretic peptide
CABG: Coronary artery bypass graft
CADSMS: Coronary artery disease self-management scale
CR: Cardiac rehabilitation
CVD: Cardiovascular disease
HADS: Hospital Anxiety and Depression Scale
HDL: High-density lipoprotein
HF: Heart failure
TG: Triglycerides
TC: Total cholesterol
LDL: Low-density lipoprotein
PCI: Percutaneous coronary intervention
PSQI: Pittsburgh Sleep Quality Index
QoL: Quality of life
SAQ: Seattle Angina Questionnaire
OR: Odds ratio
CI: Confidence interval
